# Impact of micro‐mobility‐induced step reduction in healthy adults on vascular and glycemic health: A real‐world investigation

**DOI:** 10.14814/phy2.71151

**Published:** 2026-11-05

**Authors:** Paul T. Reidy, Ashley C. Betz, Jackson P. Yeager, Yi Liu, Rachael A. Binion, Sydney Ayton, Lauren Lefkowitz, Eric Slattery, Rebecca J. Skujins, Asya Walsh, Marissa Scerbo, Kyle L. Timmerman, Kevin D. Ballard

**Affiliations:** ^1^ Department of Kinesiology, Nutrition and Health Miami University Oxford Ohio USA

**Keywords:** bioelectrical impedance, glycemic variability, micro‐mobility, pulse wave velocity, sedentary behavior

## Abstract

We investigated the effects of a 7‐day e‐scooter‐induced step reduction followed by 7 days of recovery on vascular function, body composition, and glycemic control in healthy adults. Longitudinal time series. Twelve adults (5 male and 7 women; 20.3 ± 1.8 years) completed a three‐week study: (1) baseline, (2) 7‐day step reduction via e‐scooter, and (3) 7‐day recovery. Assessments included daily steps (accelerometry), glycemic control (continuous glucose monitoring), body composition (bioelectrical impedance), and arterial stiffness (pulse wave velocity [cf‐PWV]). Steps decreased by ~33% during Reduced Activity (10,466 ± 2872 to 6965 ± 3408 steps/day; *p* < 0.05) and returned to baseline (9103 ± 2896) during Recovery. Step reduction decreased lean mass and increased glycemic variability. While some lean mass recovered, glycemic variability remained elevated during Recovery. cf‐PWV was unchanged during step reduction but decreased by 0.4 m/s after Recovery. Short‐term, modest physical activity reduction impairs glycemic control and reduces lean mass. Rapid recovery of lean mass contrasts with persistent increases in glycemic variability, suggesting residual metabolic impacts. Resuming normal activity may improve vascular function via reduced arterial stiffness.

## Graphical abstract

This study investigated the physiological effects of a modest, 7‐day reduction in daily step count induced by real‐world micro‐mobility device (e‐scooter) use in healthy adults. The forced sedentary behavior led to a significant 33% decline in daily physical activity, which acutely impaired free‐living glycemic control and increased glycemic variability. However, these negative metabolic changes were successfully reversed and metabolic health was restored following a subsequent 7‐day recovery week of normal activity.

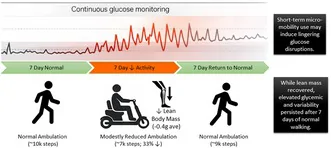

## 1 INTRODUCTION

Regular physical activity (PA) promotes and protects physical and mental health and is essential for preventing chronic disease. However, according to recent data from the World Health Organization, approximately 31% of adults globally (~1.8 billion people) and 80% of adolescents did not meet the recommended physical activity levels in 2022, highlighting an increasingly persistent and widespread shortfall of habitual PA. Together, this suggests a continued pattern of reduced PA, which is concerning as inactivity has been linked to leading chronic diseases such as cardiovascular disease, strokes, cancer, diabetes, and obesity (Anderson & Durstine, 2019; Booth et al., 2012; CDC, 2024).

As rates of PA decline in the US (CDC, 2022), various modes of electric transportation have become increasingly available. Electric scooters (e‐scooters) have gained popularity since 2017, following the introduction of e‐scooter sharing programs in more than 65 US cities (Glenn et al., 2020; Kobayashi et al., 2019). These sharing programs have provided populations with easy access to e‐scooters, thereby increasing the availability of electric transportation and reducing active transportation practices such as walking, running, or cycling (Cubells et al., 2023). A recent survey found that 43.5% of e‐scooter users reported replacing active‐transportation habits with inactivity, stating they would have walked if e‐scooters were unavailable (Glenn et al., 2020). In an increasingly sedentary society, readily accessible electric devices, such as e‐scooters, pose a micro‐mobility issue that continues to contribute to a decline in ambulatory activity.

Physical inactivity is associated with an increased risk of developing chronic diseases and metabolic dysfunction due to adverse physiological changes, including weight gain, altered blood lipids and fat deposition, loss of metabolic flexibility, musculoskeletal decline, and hormonal, inflammatory, and cardiovascular impairments (Paing et al., 2018). Using step reduction models, decreases in ambulatory physical activity by ≥60% result in deleterious health effects such as altered body composition and reductions in insulin sensitivity (Breen et al., 2013; Krogh‐Madsen et al., 2010; Olsen et al., 2008), glycemic control (Mikus et al., 2012; Paing et al., 2018), cardiorespiratory fitness (Bowden Davies et al., 2018), and vascular function (Krogh‐Madsen et al., 2010; Olsen et al., 2008; Reynolds et al., 2015). While large step reductions (≥60%) have been shown to elicit cardiometabolic dysfunction (Booth et al., 2017; Bowden Davies et al., 2018; Krogh‐Madsen et al., 2010; Olsen et al., 2008), limited data have examined the effect of a more modest step reduction on cardiometabolic health indices. The present study investigated a step reduction model that is more applicable to reductions in PA seen with lifestyle choices, thus increasing generalizability. Providing assistive electric transportation via an e‐scooter enables participants to maintain a lower level of activity with fewer adjustments to their daily schedules. Altogether, this study may be more ecologically valid and representative of the experiences of a larger portion of the population.

### 1.1 Study aim

The primary aim of this study was to investigate the effect of a 7‐day modest step reduction via the use of e‐scooters on cardiometabolic outcomes. The secondary aim of this study was to determine if the cardiometabolic changes could be recovered following 7 days of return to baseline PA. We hypothesized that 7 days of modest step reduction would negatively affect blood glucose regulation, vascular function, and body composition, with partial or full recovery of these adverse cardiometabolic changes following the resumption of baseline PA levels.

## 2 METHODS

### 2.1 Participants and ethical approval

Healthy males and female adults (age range = 18–40 years) with self‐reported walking >20 min/day and >7000 steps/day were recruited across the Miami University campus and Oxford, OH community through the use of flyers and word of mouth (Figure S1). Because the study aimed for a modest step reduction, participants were excluded if they did not adhere to the protocol or failed to reduce their average weekly step count by more than 1500 steps/day during the reduced physical activity week. Volunteers completed a pre‐screening form consisting of the Physical Activity Readiness Questionnaire for Everyone (2014 PAR‐Q+) and the International Physical Activity Questionnaire (IPAQ) to determine eligibility for participation. All participants provided written consent, and the Miami University Institutional Review Board approved the study under protocol #02082r, which is in accordance with the Declaration of Helsinki.

### 2.2 Study design

This exploratory/pilot study was conducted over three consecutive weeks using a longitudinal, quasi‐experimental design (Figure 1). During the Reduced Activity week (Day 0 to Day 7), participants were provided with an e‐scooter (Fucare H3, Rancho Cucamonga, CA) or (Ancheer IPX5, Way Cary, NC) and instructed to use it as a substitute for walking. Participants returned the e‐scooter on Day 7 and were asked to resume their daily activities during the Recovery week (Day 7 to Day 14). Across the three‐week study period, free‐living interstitial glucose, physical activity, and dietary intake were tracked. Participants visited the laboratory four times for standardized testing: on Day −7, 0, 7, and 14. On Day −7, participants provided informed consent, completed questionnaires, and were fitted with a continuous glucose monitor (CGM) and accelerometer (see below). On Days 0, 7, and 14, we assessed cardiorespiratory fitness, body composition, and vascular function, and collected a fasted blood sample.

**FIGURE 1:**
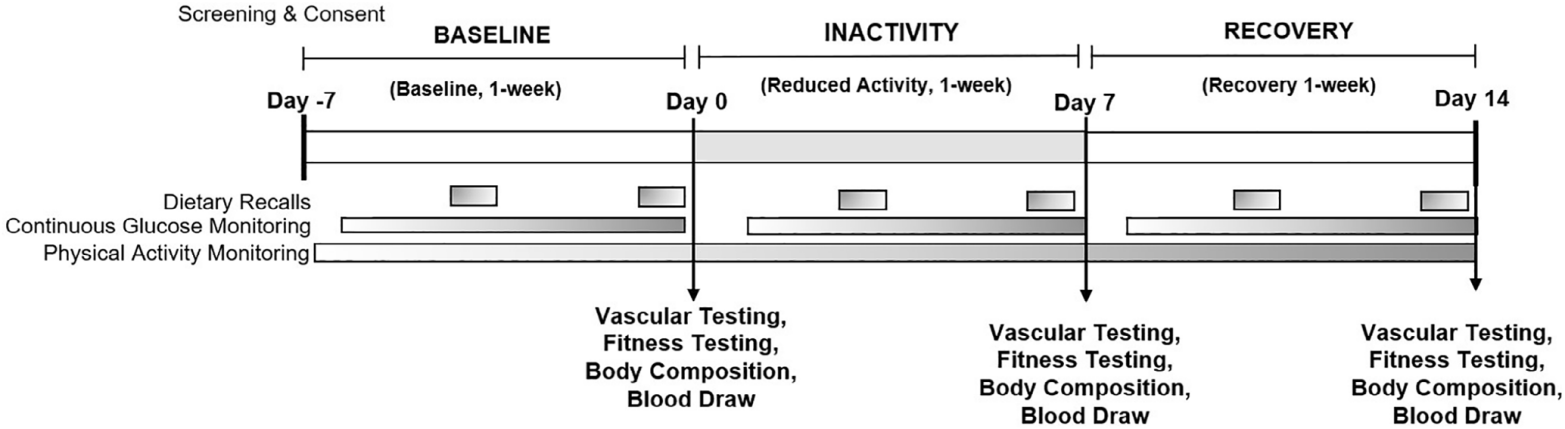
Experimental study design. Schematic representation of the 21‐day longitudinal study protocol. The study consisted of three consecutive 7‐day phases: Baseline (Days 1–7): Participants maintained habitual physical activity and dietary patterns. Reduced Activity (Days 8–14): A 7‐day intervention where participants utilized electric scooters (e‐scooters) to achieve a modest reduction (~33%) in daily step counts. Recovery (Days 15–21): A 7‐day period where participants returned to their baseline physical activity levels. Laboratory Assessment Points (T1, T2, T3): Testing was conducted at the end of each week (Days 7, 14, and 21). Assessments included bioelectrical impedance analysis (BIA) for body composition, carotid‐femoral pulse wave velocity (cf‐PWV) for arterial stiffness, and fasting blood draws for metabolic markers. Continuous glucose monitoring (CGM) and accelerometry were performed throughout the duration of the study to assess free‐living glycemic control and daily step counts, respectively.

### 2.3 Study days

Prior to each laboratory visit, participants were instructed to abstain from alcohol consumption for 48 h, from exercise for 24 h, and from caffeine intake for 12 h. Further, participants were asked to eat a typical dinner the prior evening and not consume any foods and beverages, except water, after 2200 h. Participants were instructed to wear the same loose‐fitting clothing for each study visit.

Day −7: After informed consent was obtained, participants were instructed to complete physical activity questionnaires (e.g., PAR‐Q+, IPAQ) to confirm eligibility. Participants received an orientation and safety course on e‐scooter use. Height, body mass, and composition were measured using a wall‐mounted stadiometer and bioelectrical impedance analysis (BIA). The CGM (Freestyle Libre Pro, Abbott, Alameda, California) was placed on participants' upper, posterior arm and activated. Accelerometers (activPAL, PAL Technologies Ltd., Glasgow, Scotland) were activated and placed on participants' right anterior thigh and secured with water‐resistant adhesive tape.

Days −7, 0, 7 and 14: The day prior to the participant's arrival, a pre‐visit checklist was filled out via Google Forms by the participant to remind them of the standardization of protocols (overnight fasting, proper fluid intake, avoidance of interfering medications). Next, BIA was performed to assess body composition. Vascular function was assessed, and whole blood was collected via venipuncture. Next, cardiorespiratory fitness was assessed using the YMCA submaximal cycle ergometer test.

### 2.4 Aerobic fitness

Cardiorespiratory fitness was estimated via a YMCA submaximal cycle ergometer test (LODE Corival Ergometer, Groningen, The Netherlands) (Golding, 2000). Heart rate (Polar T31 HR monitor (Kempele, Finland)) and 6–20 scale rating of perceived exertion (RPE) were monitored every minute during the test and during a 5‐min cooldown period.

### 2.5 Body composition

BIA (for visceral fat, lean and muscle mass estimates) was performed after the bladder was voided to assess body composition. Body composition (total lean mass (TLM), lean leg mass (LLM), % body fat (%BF), visceral adipose tissue (VAT), and skeletal muscle mass (SMM)) was measured using bioelectrical impedance (InBody 770, Cerritos, CA).

### 2.6 Dietary analysis

Dietary analyses were conducted using the Automated Self‐Administered 24‐h Dietary Assessment Tool (ASA24), developed by the National Institutes of Health (NIH). The ASA24 website allows participants to record all food and drinks consumed over the previous 24 h. Each participant was assigned a unique login and instructed to record two dietary recalls per week: one on a weekday and one on a weekend day. P. Data reports were generated from ASA24 to assess the composition of participants' daily dietary intake (i.e., protein, carbohydrate, fat, sodium, micronutrients, total added sugar).

Weekly dietary intake was calculated using the Healthy Eating Index (HEI) into a standardized score to assess nutritional quality and adequacy. Food groups are individually scored based on the cup equivalence of foods consumed from a range of 0–5. These individual scores are added together to produce a combined HEI score. Total HEI scores over 80 imply consumption of a ‘good’ diet, scores between 51 and 80 indicate a ‘need for improvement’, and scores below 51 indicate a ‘poor’ diet.

### 2.7 CGM data analysis

CGM measurements were collected using Freestyle Libre Pro sensors and software (Abbott, Alameda, California). Participants wore a sensor for the duration of the study, with the first being placed on day −7 and a second sensor replacing it on either day 0 or day 7. Data from the first day of wear time were excluded due to the potential for lower, less accurate results as the sensor stabilizes (FreeStyle Libre Providers, 2023; Shah et al., 2019). Sensors were placed on the back of the arm on the opposite side of the body to the side the participant tended to sleep on, after the skin was cleaned with an alcohol wipe. To prevent falsely altered sensor glucose values, participants were instructed to avoid using any products that contain salicylic acid, taking vitamin C supplements, acetaminophen, and lying directly on the sensor (per manufacturer documentation). The sensors collected data every 15 min, beginning 1 h after installation. Data were exported and underwent a quality check before analysis. The quality check included: any CGM readings <50 mg/dL during sleep time as defined by accelerometer lying down; 3 or more consecutive readings of <50 mg/dL flanked by CGM readings of >80 mg/dL within 30 min before or after the string; 3 or more consecutive readings in which readings of >200 mg/dL are interspersed with readings of <70 mg/dL.

Data points were either excluded if two or more sequential values met the criteria, or averaged using surrounding data points if only one value met the criteria. Quality‐checked data was then analyzed using R software with the iglu version 3.0.0. Data from iglu includes active wear time percent, coefficient of variation (CV), mean amplitude of glycemic excursions (MAGE), time below range of 70 mg/dL (TBR <70), time in range of 70–120 mg/dL (TIR 70–120), standard deviation (SD), median, mean, and area under the curve (AUC).

### 2.8 Vascular data analysis

Pulse wave analysis (PWA) and carotid‐femoral pulse wave velocity (cf‐PWV) were measured on days 0, 7, and 14 using the SphygmoCor XCEL system (AtCor Medical, Naperville, IL). Brachial pulse waveforms were recorded twice following 5 min of seated rest to calculate aortic systolic (SBP) and diastolic pressure (DBP), mean arterial pressure (MAP), aortic pulse pressure (PP), augmentation pressure (AP), augmentation index (AIx), and heart rate normalized AIx (AIx75). Carotid‐femoral PWV was measured twice following 10 min of supine rest (Alessio et al., 2024).

### 2.9 Blood lipid and insulin analyses

Whole blood was collected following an overnight fast for the determination of total cholesterol (TC), high‐density lipoprotein‐cholesterol (HDL‐C), low‐density lipoprotein‐cholesterol (LDL‐C), and glucose (Alere Cholestech LDX, San Diego, CA). Serum insulin was measured via ELISA following the manufacturer's instructions (Millipore EZHI‐14K, St. Louis, MO).

### 2.10 Activity and scooter usage tracking

On the morning of day 14, the accelerometer (activPAL, PAL Technologies Ltd., Glasgow, Scotland) was removed and data were exported and processed (PALanalysis v8.11.8.75) to calculate for each week (i.e., Baseline, Reduced Activity, Recovery) daily steps, sitting time, stepping time, primary lying time, secondary lying time, total sedentary time, activity score (i.e., MET/h per week). We excluded days with <1000 min of wear time. To track scooter usage, we created a commercial GPS tracking account (Strava Inc., San Francisco, California) with privacy set to private for each participant to log usage on their smartphone so we could collect miles, distance, and time ridden.

### 2.11 Statistical analysis

Absolute and baseline‐subtracted change data (Δ) were evaluated across the testing period. Data normality was confirmed using a Shapiro–Wilk test (*p* > 0.05). Initial analyses revealed no significant sex differences or sex‐by‐time interactions; therefore, data were collapsed across sex for all subsequent analyses. Absolute values across timepoints (Baseline, Reduced Activity, Recovery) were evaluated using a one‐way ANOVA (GraphPad Prism, Version 10.5.0). To directly assess specific pairwise hypotheses regarding intervention and recovery magnitude, change scores (Δ Reduced Activity vs. Baseline; Δ Recovery vs. Baseline) were analyzed using paired *t*‐tests. All data are presented as mean ± SD or mean ± 95% CI. Statistical significance was set at *p* < 0.05, and statistical trends were defined as 0.05 < *p* < 0.10.

## 3 RESULTS

### 3.1 Participant characteristics

Twelve (*n* = 12; 5 males and 7 females) participants met the inclusion criteria for analysis. The mean age of participants was 20.3 ± 1.8 years, a mean BMI of 21.9 ± 1.9 kg/m^2^, body fat percentage (BFP) of 20.8% ± 8.7%, lean body mass of 51.3 ± 8.8 kg, and estimated VO2peak of 41.2 ± 13.4 mL/kg/min. Baseline lipid analysis showed a mean cholesterol of 151.3 ± 28.2 mg/dL, HDL‐C of 54.1 ± 14.3 mg/dL, LDL‐C of 92.6 ± 29.3 mg/dL, and a fasted blood glucose of 88.5 ± 6.1 mg/dL.

Participants self‐reported via IPAQ engaging in physical activity at an average of 4242 ± 5525 MET‐minutes per week (across all activities) and spent an average of 6.2 ± 3.5 h per day in sedentary activities (sitting time). ActivPal data showed sitting time was 8.1 ± 2.6 h per day.

### 3.2 Physical activity

During the Reduced Activity week, GPS tracking data revealed the following total weekly scooter usage: 22.6 ± 14.8 rides, 141.8 ± 57.9 min, and 28.6 ± 13.6 km. This resulted in an average of 2.9 ± 1.8 uses, 18.4 ± 7.1 min, and 3.8 ± 1.8 km per day for each participant. Scooter use did not differ between male and female adults (data not shown).

Compared to Baseline, total daily steps, activity score (MET/h), and total stepping time decreased (*p* < 0.001, 0.001, and 0.013, respectively), and total sitting time increased (*p* = 0.020) during the Reduced Activity week (Table S1 and Figure 2). No differences were found between the Baseline and Recovery weeks for average total daily steps or activity score (Table S1 and Figure 2), or between male and female adults (Table S2). Yet, trends (*p* < 0.1) for incomplete recovery of steps, sitting time, and activity score between Baseline and Recovery were observed.

**FIGURE 2:**
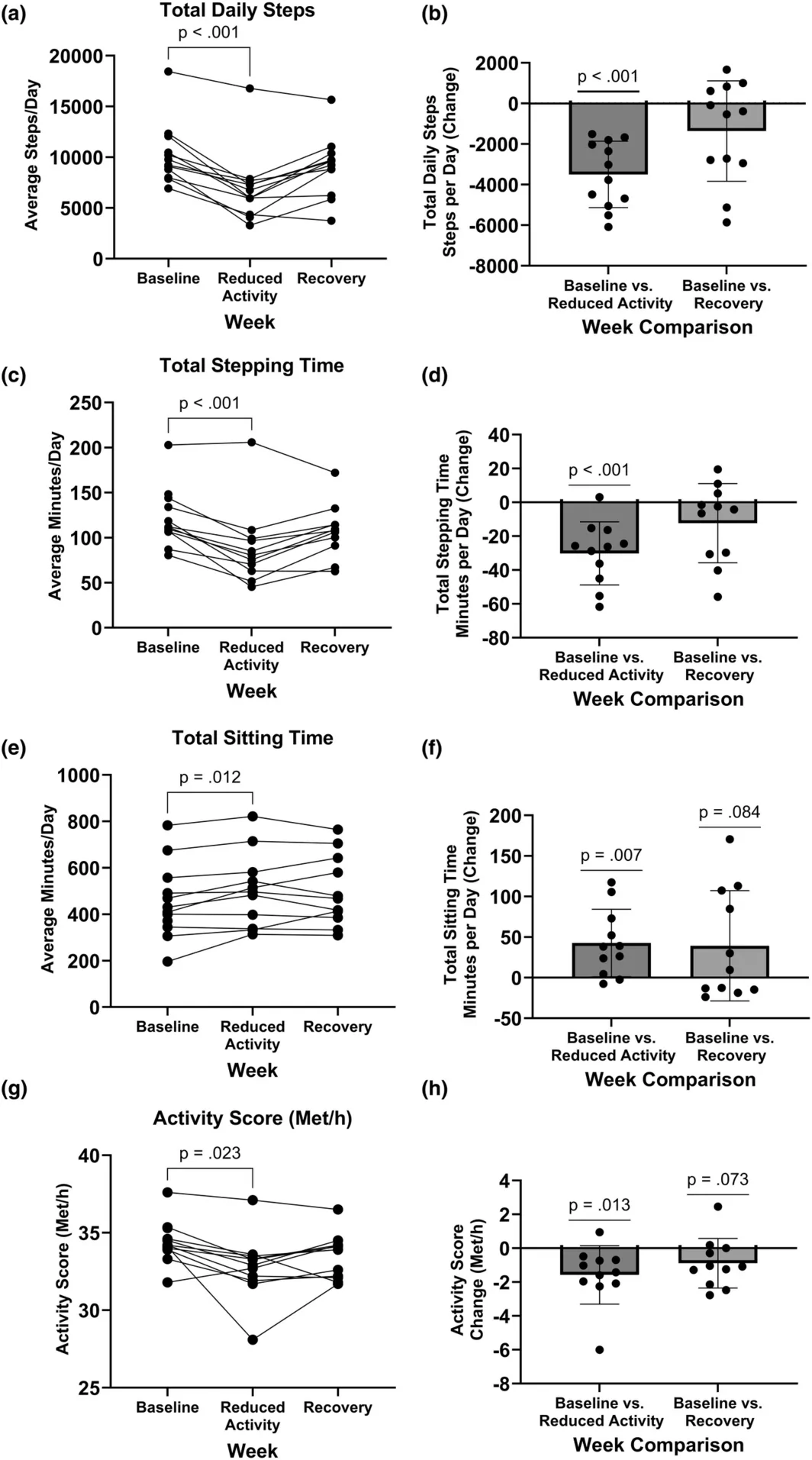
Accelerometry weekly average physical activity metrics of total daily steps per day (a, b), total daily minutes of stepping time (c, d), and Activity Scote (Met/h) (g, h) total daily minutes of sitting time (e, f). Data from young healthy adults are shown as absolute values (a, c, e) during 1 week of normal activity (Baseline), followed by 1 week of modest step reduction (Reduced Activity), and then 1 week of return to regular activity (Recovery) and the changes (b, d, f, h) from Baseline versus Reduced Activity and Baseline versus Recovery. Data are Mean ± SD. Change is compared to 0.

### 3.3 Dietary intake

Dietary protein, carbohydrate, fat, added sugar consumption, and Healthy Eating Index (HEI) were unchanged across the 3‐week study period and did not differ between males and females, so data was collapsed (Table S3).

### 3.4 Fasting blood lipids, glucose, serum insulin, and HOMA‐IR

Fasting blood TC, LDL‐C, glucose, serum insulin, and HOMA‐IR were unchanged across the 3‐week study period, whereas HDL‐C was lower (*p* < 0.046) during Recovery compared to Baseline (Table S4).

### 3.5 Body composition and VO_2_peak

BMI, %BF, VAT, TLM, and SMM showed no main effects (Table 1). Total lean mass decreased (*p* = 0.038), and leg lean mass increased (*p* = 0.020), 0.20 kg (0.04, 0.36) from Reduced Activity to Recovery. Trends (*p* < 0.1) for decreased leg lean mass and skeletal muscle mass between Baseline and Reduced activity were observed (Table 1). Estimated VO_2_peak calculated from the YMCA submaximal cycle test was 41.3 ± 14.0 mL/kg/min at Baseline, 37.2 ± 8.5 mL/kg/min at Reduced Activity, and 39.7 ± 8.1 mL/kg/min at Recovery. A concerning change from Baseline to Reduced Activity of −4 mL/kg/min (−9.2, 1.2) did not reach statistical significance, but was restored by an increase from Reduced Activity to Recovery of 2.4 mL/kg/min (0.4, 4.3).

**TABLE 1:** Body composition metrics of body fat %, visceral fat (cm^2^), total lean mass (kg), leg lean mass (kg) and skeletal muscle mass in young healthy adults are shown as absolute values during 1 week of normal activity (Baseline), followed by 1 week of modest step reduction (Reduced Activity), and then 1 week of return to regular activity (Recovery) with the changes from Baseline versus Reduced Activity and Baseline versus Recovery.

	Absolute			Change	
	Baseline	Reduced activity	Recovery	Baseline versus reduced activity	Baseline vs. recovery
BMI (kg/m^2^)	21.94 ± 1.88	21.82 ± 1.92	21.87 ± 1.82	−0.12 (−0.27, 0.03)	−0.08 (−0.24, 0.08)
Body fat (%)	20.8 ± 8.7	21.2 ± 8.4	21.2 ± 8.6	0.37 (−0.2, 0.93)	0.37 (−0.20, 0.94)
Visceral fat (cm^2^)	56.16 ± 29.76	56.55 ± 28.77	56.5 ± 27.86	0.39 (−2.38, 3.16)	0.34 (−2.10, 2.78)
Total lean mass (kg)	51.26 ± 8.79	50.73 ± 8.41	50.83 ± 8.59	−0.53 (−1.02, −0.03)*	−0.43 (−1.05, 0.20)
LLM (kg)	15.97 ± 2.95	15.8 ± 2.8	16 ± 2.81	−0.17 (−0.35, 0.02)^#^	0.03 (−0.15, 0.22)
SMM (kg)	28.52 ± 5.34	28.23 ± 5.13	28.25 ± 5.29	−0.28 (−0.58, 0.01)^#^	−0.27 (−0.61, 0.08)

*Note*: Absolute values are reported as Mean ± SD. Change Data values are reported in mean (95% confidence intervals).

* *p* < 0.05.

^#^ *p* < 0.1.

### 3.6 Vascular function

cf‐PWV was lower (*p* = 0.031) at the end of the Recovery week than at Baseline (Table S5). No other changes in vascular function were observed.

### 3.7 Glycemic control

Free‐living interstitial glucose control metrics are shown in Figure 3 and Table S6. Compared to Baseline, 24‐h interstitial glucose Median glucose, 24‐h interstitial glucose Mean Amplitude of Glycemic Excursions (MAGE), and 24‐h Mean interstitial glucose were increased (*p* = 0.034, 0.026 and 0.029, respectively) following the Reduced Activity week. The increase in 24‐h interstitial glucose MAGE persisted (*p* = 0.020) following the Recovery week compared to Baseline. No effects were observed for 24‐h interstitial glucose %Coefficient of Variation (%CV), 24‐h interstitial glucose Time Below Range (TBR) (%), or 24‐h interstitial glucose Time in Range (TIR) (%). Recovery decreased compared with baseline for 24‐h TBR (*p* = 0.017). No sex differences were observed in the absolute values of the CGM metrics in our sample of five males and seven females (data not shown).

**FIGURE 3:**
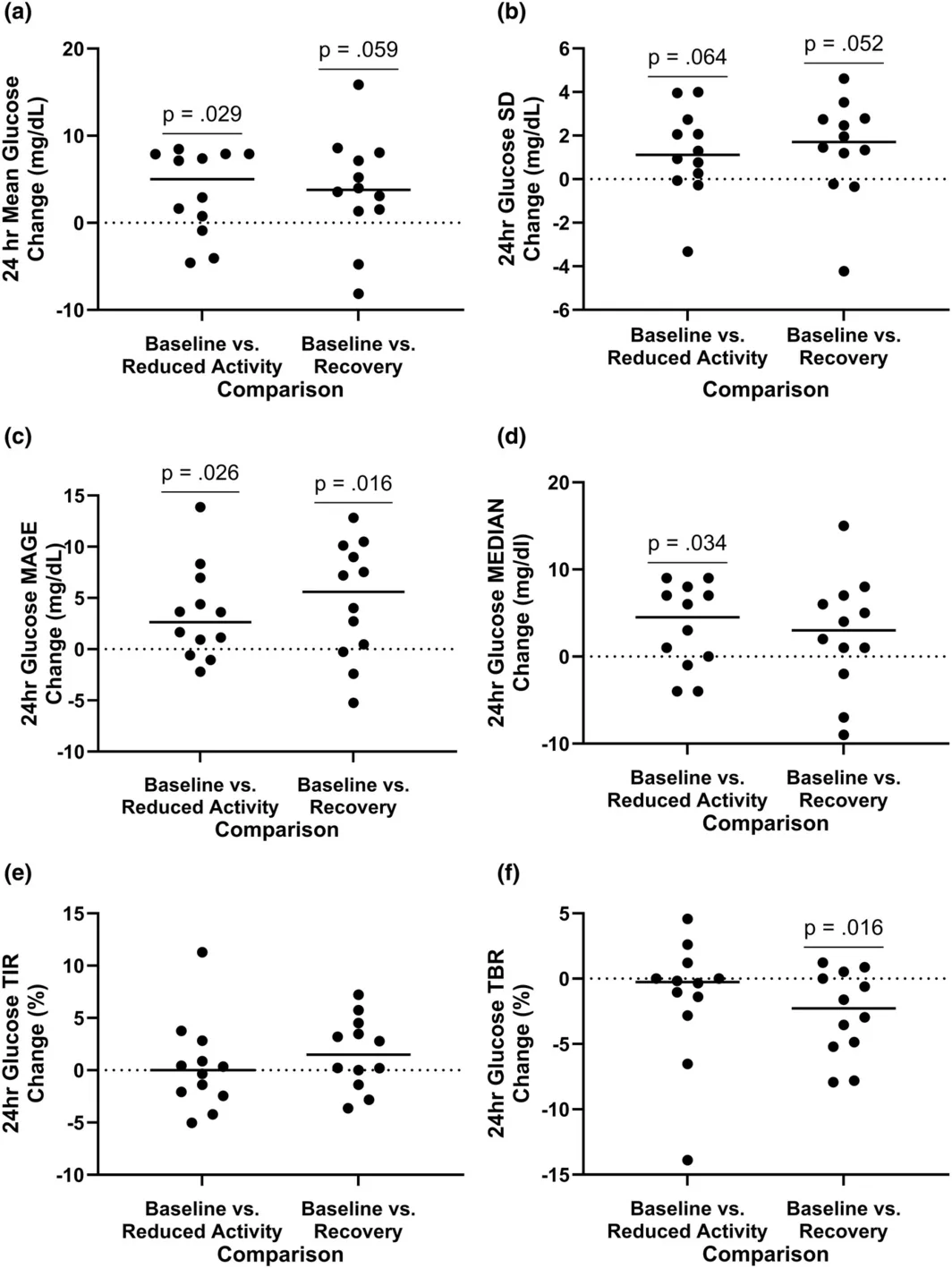
Free‐living continuous glucose monitoring metrics of 24 h glycemic control (Glucose Mean (a), Glucose SD (b), MAGE (c), Glucose Median (d), Glucose TIR (e) and Glucose TBR (f)). Data from young healthy adults are shown as individual data points and mean (black line) for the changes from Baseline versus Reduced Activity and Baseline versus Recovery. Change *p*‐values are compared to 0.

## 4 DISCUSSION

Short‐term step‐reduction studies in healthy adults have shown adverse effects on glucose tolerance, body composition, and vascular function (Bowden Davies et al., 2019; Bowden Davies et al., 2021). However, these studies reduced daily steps to levels (~70%–85%) that are less generalizable to what the population commonly experiences. Thus, our aim in this exploratory/pilot study was to explore the effects of a more modest and ecological step‐reduction model facilitated by the use of inactive transportation (i.e., e‐scooter) on glucose control, body composition, and vascular function in healthy adults.

We showed that access to an e‐scooter for 1 week decreased average daily steps by approximately 33% (~3500 steps/day) and total stepping time by approximately 30 min/day, along with a 43 min/day increase in sitting time, compared to the Baseline week. Physical activity was partly returned to baseline after a week of normal ambulation. We observed a trend (*p* < 0.1) for a ~39 min/day increase in sitting time and only partial recovery of daily steps (13%, ~1362 steps/day less than Baseline). Although this pattern was not consistent across all participants, this may have contributed to incomplete restoration of glycemic control. The short‐term reduction in PA increased 24‐h glucose load (Mean, Median) and variability (SD and MAGE), with some CGM measures remaining elevated even after a week of return to regular activity. Additionally, the transient reduction in PA resulted in a ~1% decrease in total lean mass but did not affect estimated cardiorespiratory fitness, blood lipids, glucose levels, insulin resistance, or vascular function.

Transient step reduction induces negative changes in glucose control in both young adults (Bowden Davies et al., 2021; Knudsen et al., 2012; Olsen et al., 2008) and older adults (Reidy et al., 2018; Bowden Davies et al., 2019; McGlory et al., 2018). These studies have shown a decrease in glucose tolerance (Bowden Davies et al., 2018; Bowden Davies et al., 2021; Breen et al., 2013; Knudsen et al., 2012; Krogh‐Madsen et al., 2014; McGlory et al., 2018; Olsen et al., 2008), increased insulin resistance (Credeur et al., 2019; Golding, 2000; Reidy et al., 2019), and/or increased mean 24‐h glucose (Krogh‐Madsen et al., 2014) after 7–14 days of reduced PA. Only three studies are similar in length to the present study. Two of which have shown 3–5 days (Mikus et al., 2012; Reynolds et al., 2015) of step reduction increased postprandial glucose and free‐living glycemic variability. Thus, transient step reduction clearly worsens glycemic control within a week.

Previous studies enrolled participants accumulating 7000–12,000 steps/day at baseline then reduced daily steps by ~50%–85% for 3–7 days, aiming for a reduction >5000 steps per day. We demonstrated a modest, more generalizable step reduction of ~33% (~3500 steps/day) for 7 days, accompanied by a reduction in stepping time and increased sitting time, and impaired free‐living glycemic control measured by continuous glucose monitoring. Interestingly, previous research suggests that the magnitude of step reduction does not correlate with the extent of changes in insulin sensitivity in old (Reidy et al., 2018) and young (Krogh‐Madsen et al., 2010) adults. This suggests a potential threshold of step reduction that occurs to reduce glycemic control. That pattern, combined with our data, indicates that even a modest step reduction (33%) is enough to elicit changes in glycemic control, and if a threshold exists, it would be less than a 33% reduction, at least in our population studied.

As most step reduction studies use OGTTs, an additional novelty of our study was the use of an ecologically valid assessment of free‐living glucose control with CGMs. Only four previous studies reported the use of CGMs (Krogh‐Madsen et al., 2014; Mikus et al., 2012; Reynolds et al., 2015; Winn et al., 2019) over three (Mikus et al., 2012), five (Reynolds et al., 2015), ten (Winn et al., 2019) and 14 days (Krogh‐Madsen et al., 2014) of step reduction, and only one study reported use in the recovery period following step reduction, and that was for only 1 day (Reynolds et al., 2015). Mikus et al. and Reynolds et al. used CGMs during 3 and 5 days of step reduction, respectively, and demonstrated greater postprandial glucose levels with increased glycemic variability (Mikus et al., 2012; Reynolds et al., 2015). Krogh‐Madsen et al. (2014) studied an overfeeding active group and an overfeeding step reduction group and found that the overfeeding step reduction group increased the 24‐h mean glucose and glycemic variability, whereas their control group did not (Krogh‐Madsen et al., 2014). No group has examined 7 days of step reduction with CGMs without adding a dietary intervention to the design. We report that 7 days of modest step reduction increased 24‐h glucose load (Mean, Median) and variability (SD and MAGE). In contrast to the three aforementioned studies and our own, the 2019 Winn et al. study demonstrated a decrease in 24‐h mean glucose in their inactive group compared to the control group, and they did not report any glycemic variability data (Winn et al., 2019). An important distinction is that they initiated a ~400 kcal/day deficit to account for the reduced energy expenditure during their 10‐day step reduction, showing that glycemic load (CGM) and control (OGTT) are improved or preserved (Winn et al., 2019). It is interesting to hypothesize that in our study a likely energy surplus (same intake + reduced expenditure) may have occurred as a plausible real‐world mechanism driving the metabolic changes.

The recovery of glycemic control following a period of reduced activity has been less studied, yet it is quite likely a key period in the development of cardiometabolic disease. Only the Reynolds et al. (2015) study reported using CGM assessment during the recovery period, and they did so only for the first day of recovery to demonstrate a maintained increase in post‐prandial glucose, but not in CONGA‐2 (Reynolds et al., 2015). Yet, visual inspection of their one representative minute‐by‐minute blood glucose measures obtained via CGM also suggested that the impairments in free‐living glycemic control were still apparent during that time. They did not report 24‐h mean glucose or other common glycemic control measures. In contrast, our CGM data extends beyond 1 day of recovery from step reduction and shows that some metrics of glucose control remain elevated. These include increased 24‐h MAGE (a metric of variability), a trend for increased 24‐h mean glucose, and 24‐h glucose SD. Interestingly, participants also demonstrated less time below the range (<70 mg/dL). It is essential to note that, despite a 3–4 mg/dL increase in 24‐h mean glucose compared to baseline, all participants remained well within the range for typical metrics of glucose load and variability for healthy non‐diabetics (Shah et al., 2019). Although they did not become clinically unhealthy, the direction of change and lack of return to baseline of their glucose control elicits several questions: how long after the end of reduced activity does the glucose control recover, and with that, do repeated bouts of reduced activity and recovery accumulate over time to shift participants closer to and into non‐healthy thresholds of glycemia, does the duration between reduced activity and recovery bouts impact this and are specific populations more affected than others? The implications for failed recovery are profound, as the inability to recover may contribute to the acceleration of metabolic disease. The clinical relevance lies not in the absolute change in healthy people, but in the system's failure to return to baseline, which has implications for repeated bouts of inactivity (e.g., hospitalization or lifestyle cycling).

Previous research has demonstrated that recovery from step reduction leads to the restoration of glucose control in healthy participants (Bowden Davies et al., 2021; Knudsen et al., 2012). Other populations may not recover metabolic function following reduced activity, such as those with first‐degree relatives with metabolic disease (Bowden Davies et al., 2018) or those who already present with comorbidities (McGlory et al., 2018). While this recovery period is much less studied than the reduced activity periods, there is some evidence suggesting that following a reduced activity period, individuals may not return to their baseline level of activity (Reidy et al., 2018). The combination of this evidence is disconcerting as the populations that have been shown to have attenuated recovery are more likely to be hospitalized and have their activity reduced, such as older adults or those with comorbidities such as diabetes and obesity. The catabolic crisis model proposed by English and Paddon‐Jones (English & Paddon‐Jones, 2010) effectively models this idea, but from a muscle, rather than metabolic, perspective; the impaired recovery from periods of reduced activity results in a lower baseline for subsequent bouts of reduced activity, thereby compounding a negative impact on overall health. The majority of these step‐reduction studies have resulted in activity levels that decreased by more than 75%. More research is needed to determine whether modest reductions in activity levels have detrimental effects and whether the resulting deficits are recoverable.

Body composition changes following short‐term step reduction are generally minor and secondary to glycemic shifts (Krogh‐Madsen et al., 2010). We observed no change in fat mass, but noted transient reductions in total (−0.52 kg) and leg lean mass (−0.16 kg), which fully restored upon recovery (with a 0.2 kg supercompensation in leg lean mass likely reflecting fluid shifts). These modest, reversible changes align with previous short‐term interventions in young adults (Reidy et al., 2018; Bowden Davies et al., 2021; Knudsen et al., 2012).

To our knowledge, no studies have investigated the effects of decreases in daily PA (and resumption of normal PA) on central arterial stiffness. Carotid‐femoral PWV was unchanged during the Reduced Activity week, but was 0.4 m/s lower (indicating improved central arterial stiffness) at the end of the Recovery week compared to Baseline, reflecting a more traditional benefit of becoming active. Our data suggest that resuming normal PA after a week of reduced PA is associated with improved central arterial stiffness in healthy individuals. In contrast to our findings, studies in healthy adults show that a transient reduction in physical activity (i.e., 3 h of uninterrupted sitting) negatively affects central arterial stiffness as shown by an increase in cf‐PWV (Credeur et al., 2019; Evans et al., 2019). Future research is needed to determine differences in the vascular effects of short‐term changes in PA in those at increased cardiometabolic disease risk. These studies would provide support for the development of interventions to decrease periods of reduced PA and improve cardiovascular health.

### 4.1 Limitations

First, the 1‐week intervention duration precludes extrapolating these findings to chronic step reduction, though it was sufficient to demonstrate rapid glycemic impairment. Second, while dietary intake was unconstrained to capture real‐world behavioral adjustments, food logs confirmed no systematic changes in caloric intake across study weeks. Third, our healthy sample limits generalizability to populations with baseline metabolic dysfunction. Lastly, while BIA estimates of lean mass are sensitive to acute hydration shifts, standardized testing conditions were maintained across weeks to minimize measurement variability.

### 4.2 Practical implications

- Modest reductions in daily steps (facilitated by micro‐mobility technology) can lead to transient decreases in lean body mass, though these changes appear reversible with a return to normal ambulation.
- Negative alterations in glycemic variability and glucose control occur even with modest step reduction and may persist longer than body composition changes, suggesting a “lag” in metabolic recovery.
- Public health and clinical interventions should prioritize reducing sedentary transit (e.g., e‐scooters) for short‐distance travel to maintain metabolic health and daily physical activity levels.
- Practitioners should recognize that even small‐scale, modern behavioral shifts in movement can measurably affect vascular stiffness and metabolic stability.

### 4.3 Conclusions

Our study demonstrated that a modest, ecologically valid reduction in daily steps (~33%) facilitated by inactive transportation (i.e. micro‐mobility) negatively affected free‐living glycemic control and lean mass in healthy adults. Importantly, our comparison is against established literature on larger step reductions, and the current findings define a new, lower threshold for metabolic changes. While the reductions in lean mass appeared transient and reversible, the incomplete recovery of glycemic variability and glucose load suggests a lingering, yet minimal, metabolic impact that extends for several days beyond the period of reduced activity. These findings indicate that even minor deviations in physical activity disrupt metabolic homeostasis, and future research should determine whether incomplete recovery between repeated bouts of reduced activity contributes to the development of cardiometabolic disease. These data underscore how sensitive human physiology is to even subtle reductions in ambulation and highlight the importance of maintaining regular physical activity to preserve long‐term metabolic health.

## AUTHOR CONTRIBUTIONS

**Paul T. Reidy:** Conceptualization; data curation; formal analysis; funding acquisition; investigation; methodology; project administration; supervision; visualization. **Ashley C. Betz:** Formal analysis; investigation; visualization. **Jackson P. Yeager:** Formal analysis; investigation; methodology; visualization. **Yi Liu:** Formal analysis; investigation; methodology. **Rachael A. Binion:** Formal analysis; investigation; software. **Sydney Ayton:** Investigation. **Lauren Lefkowitz:** Investigation. **Eric Slattery:** Investigation; resources; software. **Rebecca J. Skujins:** Formal analysis; investigation. **Asya Walsh:** Investigation. **Marissa Scerbo:** Investigation. **Kyle L. Timmerman:** Conceptualization; investigation; methodology; project administration; resources. **Kevin D. Ballard:** Conceptualization; formal analysis; investigation; methodology; software.

## FUNDING INFORMATION

This work was supported by the Miami University College of Education, Health and Society Seed Grants, Miami University Committee for Faculty Research (P.T. Reidy) and, the Miami University Undergraduate Research Award (Y. Liu, R. Skujins, A. Betz and S. Ayton) the Miami University Undergraduate Summer Scholars Program (J. Yeager, R. Skujins, R. Binion).

## CONFLICT OF INTEREST STATEMENT

No conflicts of interest, financial or otherwise, are declared by the authors.

## ETHICS STATEMENT

We confirm that we have read the Journal's position on issues involved in ethical publication and affirm that this report is consistent with those guidelines.

## Supporting information


**Table S1:** Accelerometry weekly average physical activity metrics of total daily steps per day, total daily minutes of stepping time, and total daily minutes of sitting time in young healthy adults are shown as absolute values during 1 week of normal activity (Baseline), followed by 1 week of modest step reduction (Reduced Activity), and then 1 week of return to regular activity (Recovery), with the changes from Baseline versus Reduced Activity and Baseline versus Recovery.
**Table S2:** Accelerometry weekly average physical activity metrics of total daily steps per day, total daily minutes of stepping time, and total daily minutes of sitting time in young healthy male and female adults shown as change data from 1 week of normal activity (Baseline), the following week of modest step reduction (Reduced Activity), or the last week with return to regular activity (Recovery).
**Table S3:** Dietary intake characteristics in young healthy adults averaged across the study.
**Table S4:** Fasting blood lipids, glucose, insulin, and HOMA‐IR in young healthy adults shown as absolute values during 1 week of normal activity (Baseline), followed by 1 week of modest step reduction (Reduced Activity), and then 1 week of return to regular activity (Recovery), with the changes from Baseline versus Reduced Activity and Baseline versus Recovery.
**Table S5:** Vascular variables in young healthy adults are shown as absolute values during 1 week of normal activity (Baseline), followed by 1 week of modest step reduction (Reduced Activity), and then 1 week of return to regular activity (Recovery), with the changes from Baseline versus Reduced Activity and Baseline versus Recovery.
**Table S6:** Free‐living 24 h glucose control metrics (A) Mean Glucose (mg/dl), Median Glucose (mg/dl), Glucose SD (mg/dl), Glucose MAGE (mg/dl), Glucose CV (%), Glucose TIR (%) and Glucose TBR (%). Data from young healthy adults are shown as absolute values during 1 week of normal activity (Baseline), followed by 1 week of modest step reduction (Reduced Activity), and then 1 week of return to regular activity (Recovery) and the changes from Baseline versus Reduced Activity and Baseline versus Recovery. Data are Mean ± SD. * = *p* ≤ 0.05; # = *p* ≤ 0.1. Change is compared to 0.
**Figure S1:** Enrollment and inclusion/exclusion flow.

## Data Availability

Data will be made available upon reasonable request.
