# Supplementary material for: Impact of micro‐mobility‐induced step reduction in healthy adults on vascular and glycemic health: A real‐world investigation
Source: Physiol Rep. 2026 Nov 5;14(21):e71151. doi: 10.14814/phy2.71151 (PMC13735211; doi:10.14814/phy2.71151)
Supplement: Supplementary file 1 [file PHY2-14-e71151-s001.docx]

#### **Supplemental Table 1.** Accelerometry weekly average physical activity metrics of total daily steps per day, total daily minutes of stepping time, and total daily minutes of sitting time in young healthy adults are shown as absolute values during one week of normal activity (Baseline), followed by one week of modest step reduction (Reduced Activity), and then one week of return to regular activity (Recovery), with the changes from Baseline vs. Reduced Activity and Baseline vs. Recovery.

|  | **Absolute** | | | **Change** | |
| --- | --- | --- | --- | --- | --- |
|  | **Baseline** | **Reduced Activity** | **Recovery** | **Baseline vs. Reduced Activity** | **Baseline vs. Recovery** |
| **Average daily steps (steps/day)** | 10266±3037 | 6940±3428* | 9065±2969 | -3500 (-4542,-2459)* | -1362 (-2936,212)# |
| **Average Total Sitting Time (minutes)** | 453±160 | 501±163* | 499±152 | 43 (15,71)* | 39 (-6,85)# |
| **Average Total Stepping Time (minutes)** | 122±33 | 89±43* | 107±30 | -30 (-43,-18)* | -12 (-28,3) |
| **Average Activity Score (Met/hr)** | 34.4±1.4 | 32.8±2.1* | 33.4±1.5 | -1.6 (-2.7,-0.4)* | -0.9 (-1.9,0.1)# |

Absolute values are reported as mean ± SD. Change Data values are reported in mean (95% confidence intervals) and compared to 0. *p<0.05, #p<0.1, Baseline vs Reduced Activity.

#### **Supplemental Table 2.** Accelerometry weekly average physical activity metrics of total daily steps per day, total daily minutes of stepping time, and total daily minutes of sitting time in young healthy male and female adults shown as change data from one week of normal activity (Baseline), the following week of modest step reduction (Reduced Activity), or the last week with return to regular activity (Recovery).

|  | **Baseline vs. Reduced Activity Change** | | **Baseline vs. Recovery Change** | |
| --- | --- | --- | --- | --- |
|  | **Male** | **Female** | **Male** | **Female** |
| **Average Daily Steps (steps/day)** | -2971 (-5419,-524)* | -3878 (-5164,-2592)* | -1483 (-5030,2065) | -1276 (-3502,949) |
| **Average Total Sitting Time (minutes)** | 28 (-9,65)# | 48 (-0.1,95)# | 10 (-70,90) | 56 (-12,125)# |
| **Average Total Stepping Time (minutes)** | -43 (-118,32) | -38 (-54,-22)* | -33 (-116,49) | -17 (-41,7) |
| **Average Activity Score (Met/hr)** | -7.7 (-26.9,11.4) | -1.7 (-3.8,0.3)# | -7.5 (-26.8,11.8) | -0.8 (-2.42,0.9) |

Data are Mean (95% confidence intervals). *p<0.05, #p<0.1 vs 0.

#### **Supplemental Table 3.** Dietary intake characteristics in young healthy adults averaged across the study.

| **Kilocalories** | **Protein (g)** | **Fat (g)** | **Carbohydrates (g)** | **Added Sugars (tsp)** | **HEI (0-100)** |
| --- | --- | --- | --- | --- | --- |
| 1833 (1497, 2169) | 69 (54, 83) | 82 (66, 97) | 210 (165, 255) | 16 (11, 22) | 45 (41, 48) |

Absolute values are reported as Mean ± SD.

#### **Supplemental Table 4.** Fasting blood lipids, glucose, insulin, and HOMA-IR in young healthy adults shown as absolute values during one week of normal activity (Baseline), followed by one week of modest step reduction (Reduced Activity), and then one week of return to regular activity (Recovery), with the changes from Baseline vs. Reduced Activity and Baseline vs. Recovery.

|  | **Absolute** | | | **Change** | |
| --- | --- | --- | --- | --- | --- |
|  | **Baseline** | **Reduced Activity** | **Recovery** | **Baseline vs. Reduced Activity** | **Baseline vs. Recovery** |
| **Total Cholesterol (mg/dl)** | 151.4±28.6 | 140.6±23.9 | 145±28.5 | -10.8 (-1.1,-20.5) | -6.4 (-16.2,3.4) |
| **HDL (mg/dl)** | 54.1±14.3 | 52.9±15.1 | 46.5±17.3 | -1.2 (-7.1,4.7) | -7.6 (-0.14,-15.1)* |
| **LDL (mg/dl)** | 92.6±29.3 | 86.9±20.7 | 90.7±23.1 | -5.7 (-18.3,6.9) | -1.9 (-13.8,10) |
| **Glucose (mg/dl)** | 88.5±6.1 | 86.6±5.2 | 90.3±6.4 | -1.9 (-7.7,4) | 3.4 (-4,7.6) |
| **Insulin (μU/mL)** | 5.37±2.89 | 5.28±2.47 | 4.81±2.82 | 0.04 (-0.91,0.99) | -0.10 (-1.48,1.28) |
| **HOMA-IR** | 1.19±0.69 | 1.12±0.51 | 1.09±0.64 | -0.02 (-0.21,0.17) | 0.02 (-0.27,0.30) |

Absolute values are reported as Mean ± SD. Change Data values are reported in mean (95% confidence intervals). *p<0.05, #p<0.1. HOMA-IR, Homeostatic Model Assessment of Insulin Resistance.

#### **Supplemental Table 5.** Vascular variables in young healthy adults are shown as absolute values during one week of normal activity (Baseline), followed by one week of modest step reduction (Reduced Activity), and then one week of return to regular activity (Recovery), with the changes from Baseline vs. Reduced Activity and Baseline vs. Recovery.

|  | **Absolute** | | | **Change** | |
| --- | --- | --- | --- | --- | --- |
|  | **Baseline** | **Reduced Activity** | **Recovery** | **Baseline vs. Reduced Activity** | **Baseline vs. Recovery** |
| **SBP (mmHg)** | 111.89±5.49 | 114.11±6.01 | 110.78±6.82 | 2.22 (-0.85,5.29) | -1.11 (-4.85,2.62) |
| **DBP (mmHg)** | 67.78±7.64 | 67.33±8.23 | 65.89±7.98 | -0.44 (-3.62,2.73) | -1.89 (-6.60,2.83) |
| **MAP (mmHg)** | 80±7.65 | 81.11±7.87 | 78.67±7.71 | 1.11 (-1.75,3.97) | -1.33 (-5.22,2.55) |
| **Augmentation Pressure (mmHg)** | 1±2.74 | 1.11±2.47 | 1.11±2.42 | 0.11 (-0.86,1.09) | 0.11 (-1.90,2.13) |
| **Heart Rate (bpm)** | 70.67±13.86 | 73±13.62 | 71.33±9.67 | 2.33 (-3.39,8.06) | 0.67 (-4.39,5.72) |
| **PWV (m/s)** | 5.98±0.48 | 5.85±0.61 | 5.6±0.41 | -0.13 (-0.38,0.12) | -0.38 (-0.65,-0.12)* |

Absolute values are reported as Mean ± SD. Change Data values are reported in mean (95% confidence intervals). *p<0.05, #p<0.1

#### **Supplemental Table 6.** Free-living 24h glucose control metrics (A) Mean Glucose (mg/dl), Median Glucose (mg/dl), Glucose SD (mg/dl), Glucose MAGE (mg/dl), Glucose CV (%), Glucose TIR (%) and Glucose TBR (%). Data from young healthy adults are shown as absolute values during one week of normal activity (Baseline), followed by one week of modest step reduction (Reduced Activity), and then one week of return to regular activity (Recovery) and the changes from Baseline vs. Reduced Activity and Baseline vs. Recovery. Data are Mean±SD. * = p ≤ 0.05; #= p ≤ 0.1. Change is compared to 0.

|  | **Absolute** | | | **Change** | |
| --- | --- | --- | --- | --- | --- |
|  | **Baseline** | **Reduced Activity** | **Recovery** | **Baseline vs. Reduced Activity** | **Baseline vs. Recovery** |
| **24h Mean Glucose (mg/dl)** | 91.2±8.4 | 94.8±9.8 | 95±6.9 | 3.5 (0.4,6.6)* | 3.8 (-0.2,7.7)# |
| **24h Median Glucose (mg/dl)** | 88.4±8.6 | 91.8±10 | 91±7.8 | 3.4 (0.3,6.5)* | 2.6 (-1.6,6.8) |
| **24h Glucose SD (mg/dl)** | 14.4±2.2 | 15.6±2.2 | 15.8±2.5 | 1.2 (-0.1,2.5)# | 1.4 (-0.1,3)# |
| **24h Glucose MAGE (mg/dl)** | 35.3±7.4 | 38.7±8.6 | 40±7.2 | 3.4 (0.5,6.3)* | 4.7 (1.1,8.3)* |
| **24h Glucose CV (%)** | 15.9±2.6 | 16.6±2.6 | 16.8±3.3 | 0.7 (-0.3,1.7) | 0.9 (-0.7,2.6) |
| **24h Glucose TIR (%)** | 93.3±5.5 | 93.6±3.9 | 94.9±5.3 | 0.3 (-2.4,3.1) | 1.6 (-0.5,3.8) |
| **24h Glucose TBR (%)** | 5.8±5.9 | 4.4±4.7 | 3.2±5.1 | -1.4 (-4.5,1.5) | -2.7 (-4.7,-0.6)* |

Mage, mean amplitude of glycemic excursions; TIR, Time in Range; TBR, Time Below Range. Absolute values are reported as mean ± SD. Change Data is reported as mean (95% confidence intervals). *p<0.05, #p<0.1.

#### **Supplemental Figure 1.** Enrollment and Inclusion/Exclusion Flow.
